# Mediterranean Diet Adherence Is Associated with Lower Prevalence of Depression and Anxiety in University Students: A Cross-Sectional Study in Greece

**DOI:** 10.3390/diseases14010019

**Published:** 2026-01-03

**Authors:** Olga Alexatou, Gavriela Voulgaridou, Sousana K. Papadopoulou, Constantina Jacovides, Aspasia Serdari, Georgia-Eirini Deligiannidou, Gerasimos Tsourouflis, Myrsini Pappa, Theophanis Vorvolakos, Constantinos Giaginis

**Affiliations:** 1 Department of Food Science and Nutrition, School of the Environment, University of the Aegean, 81400 Lemnos, Greece; fnsd23003@fns.aegean.gr (O.A.); con.jacovides@gmail.com (C.J.); fnsm23028@fns.aegean.gr (M.P.); 2 Department of Physiotherapy, School of Health Sciences, International Hellenic University, 57001 Thessaloniki, Greece; gabivoulg@gmail.com; 3 Department of Nutritional Sciences and Dietetics, School of Health Sciences, International Hellenic University, 57400 Thessaloniki, Greece; souzpapa@gmail.com (S.K.P.); deligiannidoueirini@yahoo.gr (G.-E.D.); 4 Department of Psychiatry, School of Medicine, Democritus University of Thrace, 68100 Alexandroupolis, Greece; aserdari@yahoo.com (A.S.); tvorvola@med.duth.gr (T.V.); 5 Department of Medicine, School of Health Sciences, Democritus University of Thrace, 68100 Alexandroupolis, Greece; 6 Second Department of Propedeutic Surgery, Medical School, University of Athens, 11527 Athens, Greece; gtsourouflis@med.uoa.gr

**Keywords:** dietary behavior, lifestyle factors, young adults, mental health, body weight, BMI, KIDMED index

## Abstract

**Background/Objectives**: The Mediterranean diet (MD) constitutes one of the most broadly studied dietary patterns, which has been linked to the prevention of non-communicable diseases and mental health disorders. University students, a population exposed to significant psychosocial stressors and lifestyle changes, may particularly benefit from healthy eating patterns such as the MD. This study was designed to examine the potential associations of MD adherence with symptoms of depression and anxiety among Greek university students. **Methods**: A cross-sectional study was initially conducted among 7160 active university students from ten diverse geographic regions in Greece. After the enrollment procedure and the application of relevant exclusion criteria, 5191 university students (52.0% female; mean age: 21.3 ± 2.4 years) constituted the study population. MD adherence was assessed using the KIDMED index, while depressive and anxiety symptoms were evaluated using the Beck Depression Inventory-II (BDI-II) and State–Trait Anxiety Inventory (STAI-6), respectively. Sociodemographic and anthropometric data were collected for all the enrolled university students. All the questionnaires were completed by face-to-face interviews with expert personnel. **Results**: Students with low adherence to the MD were significantly more likely to report symptoms of depression (OR = 2.12; *p* ˂ 0.001) and anxiety (OR = 2.27; *p* ˂ 0.001) and to be overweight or obese (OR = 2.45; *p* ˂ 0.001) after adjustment for multiple confounding factors. Low MD adherence was also associated with male gender (OR = 0.73; *p* ˂ 0.01), living alone (OR = 0.78; *p* ˂ 0.01), smoking (OR = 0.75; *p* ˂ 0.01), low physical activity (OR = 1.84; *p* = 0.001), and poorer academic performance (OR = 0.83; *p* ˂ 0.01). **Conclusions**: Low adherence to the MD is significantly associated with increased likelihood of depression, anxiety, and excess body weight among university students in Greece. These findings underscore the importance of promoting healthy dietary habits and related lifestyle behaviors in young adult populations as a potential strategy for mental health prevention and intervention. Due to the presence of several limitations in the present study, future longitudinal and interventional studies should be performed to confirm the present findings.

## 1. Introduction

Mental disorders are among the main causes of disability worldwide across both communicable and non-communicable conditions. The Global Burden of Diseases, Injuries, and Risk Factors (GBD) study showed that anxiety and depression constitute the two most disabling disorders, with a global prevalence rate of 19.2% for depression and 16.5% for anxiety [1]. Notably, the pooled prevalence of depression and anxiety among patients coping with post-COVID-19 syndrome was estimated to be 23% based on data from 143 studies with 7,782,124 participants and 132 studies with 9,320,687 participants, respectively [2]. Given their profound impact on individual well-being and public health, mental disorders are increasingly recognized as a priority for prevention and intervention strategies [3]. Mental disorders—alongside other leading causes of disability and years lived with chronic diseases, such as cancer, obesity, and musculoskeletal disorders—have also been correlated with lifestyle behaviors. Due to this growing problem, more research is now focused on finding lifestyle factors that can be changed to improve mental health.

Among these, biological, genetic, and environmental factors, such as diet, have been identified as key determinants of mental health [4]. Numerous studies have examined associations between specific nutrients, food groups, or bioactive compounds and mental health outcomes in several age groups [5,6,7,8]. Recent research among Greek university students revealed a strong association between perceived stress, poor sleep quality, and increased prevalence of overweight and obesity, highlighting how lifestyle and mental health are tightly interconnected [9]. However, since diet is a complex and multifaceted behavior, investigating overall dietary patterns may offer a more holistic and comprehensive approach for understanding diet-related health outcomes, as well as provide public health recommendations.

University life constitutes a stressful period for students [10]. Academic pressures due to workload, coupled with the challenge of living away from the family for the first time, can heighten vulnerability to mental health problems such as anxiety and depression [11,12]. In this respect, Beiter et al. showed that the most stressed, anxious, and depressed students were transfers, upperclassmen, and those living off-campus [10]. Moreover, Bayram and Bilgel found that depression, anxiety, and stress levels of moderate severity or above were found in 27.1%, 47.1%, and 27% of university students, respectively [11]. Notably, anxiety and stress scores were higher among female students [11]. During this transitional stage, students often adopt independent dietary choices. Limited cooking skills, time constraints, and the widespread availability of fast food appear to contribute to unhealthy eating behaviors, increasing the risk of body weight gain and poor mental health, including depression and anxiety [13,14]. A study performed by Magni et al. (2022) also highlighted the psychological burden and changing lifestyle habits during academic periods, reporting a complex interaction between emotional well-being, eating behaviors, and body image [15].

The Mediterranean diet (MD) has been recognized as one of the most well-established dietary patterns associated with the prevention of non-communicable diseases (NCDs), including cardiovascular diseases and diabetes [16]. Characterized by a high intake of plant-based foods (i.e., fruit, vegetables, and whole grains), unsaturated fats and fish, along with moderate consumption of eggs and dairy and low consumption of processed and red meats, the MD provides a wide range of bioactive nutrients that may support brain function and emotional well-being [17,18]. In particular, n-3 polyunsaturated fatty acids (n-3 PUFAs), which are contained in natural oils and fatty fish, exert beneficial effects on depression [19,20]. Several pathways have been proposed for the role of n-3 PUFAs in depression, such as neuroprotective/neurotrophic mechanisms [21,22] and anti-inflammatory effects [23]. These physiological effects help explain the growing interest in the MD as a promising strategy for mental health promotion in young adults. A review article by Antonopoulou et al. (2019) highlighted the potential of MD adherence to reduce depression and stress levels and improve academic performance among university students [24].

Despite the increasing interest in the potential mental health benefits of MD, relatively few studies have specifically focused on young adult populations, such as university students. These studies mainly involved a small number of university students and did not usually simultaneously assess the impact of both depression and anxiety [24]. Moreover, most of the existing studies did not evaluate the mediating role of sociodemographic, anthropometric, and lifestyle factors in the association of MD adherence with depression and anxiety in university students. In view of the above consideration, the present study aims to cover this literature gap by examining the relationship between adherence to MD and mental health outcomes, specifically depression and anxiety, in a large number of university students from Greece, including several sociodemographic, anthropometric, and lifestyle factors as potential confounders.

## 2. Materials and Methods

### 2.1. Study Population

**Figure 1:**
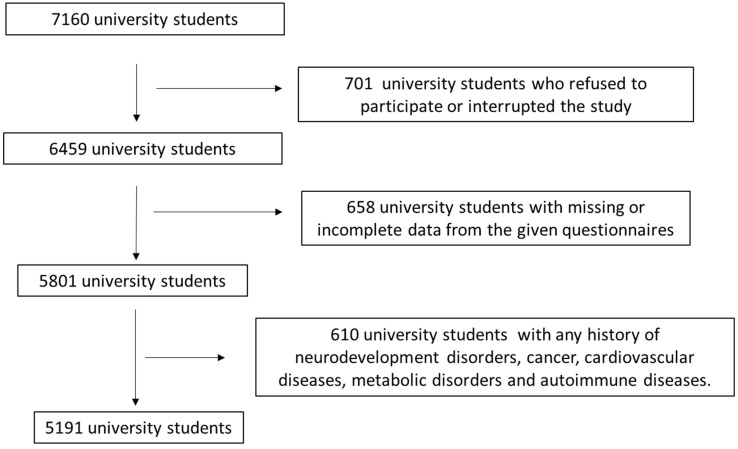
Flowchart diagram of study population enrollment.

This study initially included 7160 university students from 10 geographically diverse Greek regions, both rural and urban (Athens, Thessaloniki, Patra, Kalamata, Larissa, Kavala, Alexandroupolis, Korinthos, and South and North Aegean). The inclusion criteria for this study were any active students from the above Greek universities. The enrollment in the study was between March 2021 and October 2024. Study population enrollment was performed by visiting the local universities in the above rural and urban areas of Greece. Figure 1 shows in detail the flowchart diagram of participant assignment, including all the exclusion criteria. In total, 701 university students refused to participate or withdrew from the study. Questionnaires from 658 university students included missing or incomplete data. In addition, 610 university students had a history of chronic diseases, including cardiovascular diseases, metabolic disorders, mental illness (except for depression and anxiety), or cancer, and were excluded from participation. After the use of exclusion and inclusion criteria, 5191 university students were finally involved, leading to a final response rate of 72.5%.

Even if no a priori sample size calculation was performed, a post hoc power analysis showed that the final sample of 5191 had sufficient statistical power (>90%) to identify associations of moderate effect size (Cohen’s d ≈ 0.5; OR ≈ 1.8–2.1) at the usual significance level (α = 0.05). This sample size was considered suitable not only for identifying such associations but also in view of the study’s available resources and the reasonable 36-month enrollment interval.

The Ethics Committee of the University of the Aegean approved this survey (ethics approval code no. 21/11.10.2017, approval date: 11 October 2017) in agreement with the World Health Organization (WHO) (52nd WMA General Assembly, Edinburgh, Scotland, 2000). Informed consent by the enrolled students was provided after being presented with details about the aim of the survey. Their data was treated with strict confidentiality. The required sample size was calculated using the PS: Power and Sample Size Calculator software. Simple random sampling was performed. The computation of the power of our sample size showed a power of 88.1%. Sample size and the study’s power were computed a priori with the aim of obtaining a reliable, representative, and nationwide sample from Greece.

### 2.2. Assessment of Sociodemographic and Anthropometric Factors

Sociodemographic information was collected using structured questionnaires, which included variables such as age, gender, nationality, living arrangements, family income level, marital status of parents, smoking behavior, and employment status. Since all participants were currently enrolled in Greek universities, data regarding their academic performance and field of study were also gathered. To minimize recall bias, all the above information was obtained through individual interviews conducted by trained personnel. Family income was categorized into three groups: low (≤EUR 10,000 annually), medium (>EUR 10,000 to ≤EUR 20,000), and high (>EUR 20,000 annually). Academic performance was assessed using students’ current grade point average and categorized as follows: low (5.0–6.49), medium (6.5–8.49), and high (8.5–10.0), according to the official standards set by the Greek Ministry of Education. All the above data were collected during face-to-face interviews with qualified personnel.

Anthropometric measurements, including weight and height, were obtained at the time of data collection in order to calculate body mass index (BMI). Body weight was measured to the nearest 100 g using a Seca digital scale (Seca, Hanover, MD, USA), with participants standing barefoot. Height was assessed to be the nearest 0.1 cm using a portable stadiometer (GIMA Stadiometer 27335), also without footwear. BMI was subsequently calculated, and participants were classified as normal weight, overweight, or obese according to the WHO criteria [25,26]. Only a very small proportion of participants were classified as underweight (1.2%), with most of them presenting a BMI value close to 18.5 kg/m^2^, which was incorporated into the normal weight category.

### 2.3. Assessment of Physical Activity, Depression, and Anxiety

#### 2.3.1. Physical Activity Assessment

Physical activity was evaluated using the Greek version of the International Physical Activity Questionnaire (IPAQ), a broadly used and validated tool that captures self-reported physical activity over the previous seven days. Participants indicated the frequency and duration of their exercise habits during a typical week. The IPAQ categorizes individuals into low, moderate, or high physical activity levels based on their responses. The questionnaire has demonstrated acceptable validity and reliability across both developed and developing countries [27]. Specifically, IPAQ-Gr calculates a total physical activity score by summing the time spent on vigorous activity, moderate activity, and walking, expressed as Metabolic Equivalent of Task (MET)-minutes per week (MET·min·wk^−1^) [27]. More specifically, IPAQ uses specific truncation rules and MET, applying conversions to standardize data processing and ensuring comparability across studies. Activities exceeding 3 h (180 min) in a single bout are truncated to 180 min, permitting a maximum of 21 h of activity weekly in each category. MET values are assigned to walking (3.3 METs), moderate-intensity activity (4 METs), and vigorous-intensity activity (8 METs). These values, along with the duration and frequency of activities, are used to calculate MET-minutes, which provide a measure of total physical activity [27]. A continuous score shows total physical activity, while categorical scores provide an easy-to-understand classification, with levels defined as low (<600 MET-minutes/week), moderate (600–3000 MET-minutes/week), and high (>3000 MET-minutes/week) physical activity [27]. The Greek version of the IPAQ short form was used in the present study, which presents acceptable reliability properties in Greek young adults and shows high repeatability values for total and vigorous PA and good values for moderate and walking PA [28]. In our sample, the IPAQ scoring showed good internal consistency (Cronbach’s α = 0.84).

#### 2.3.2. Depression Assessment

Depressive symptoms were assessed using the Beck Depression Inventory-II (BDI-II), a widely recognized and extensively used psychometric tool for evaluating the severity of depression [29]. The questionnaire consists of 21 item groups that cover emotional, cognitive, and physical aspects of depression, such as hopelessness, irritability, guilt, fatigue, weight loss, and reduced sexual interest. The BDI-II has demonstrated strong internal consistency and the ability to effectively differentiate between individuals with and without depressive symptoms. It is supported by robust psychometric properties, including high levels of concurrent, content, and structural validity. Due to its reliability, ease of use, and cost-effectiveness, the BDI-II is commonly employed in both clinical and research settings worldwide [29]. A cutoff score of 20 or higher was used to identify potentially depressed university students [29]. The BDI-II Greek version was applied in the current survey, which has indicated exceptional internal reliability (Cronbach’s α = 0.93) and robust psychometric validity in both clinical and non-clinical samples [30]. In our current survey, the BDI-II exhibited high internal reliability in our sample (Cronbach’s α = 0.85), verifying its consistency for evaluating depressive symptomatology in Greek university students.

#### 2.3.3. Anxiety Assessment

Anxiety levels were measured using the six-item version of the State–Trait Anxiety Inventory (STAI-6), a brief and validated tool derived from the full State scale [31]. The STAI-6 includes six carefully selected items that maintain the reliability and validity of the original instrument. It is particularly useful in settings where time constraints or other limitations prevent the administration of the full-length version. Despite its brevity, the STAI-6 yields anxiety scores that are comparable to those produced by the complete inventory, making it suitable for both clinical and research applications [31]. A cutoff score of 13 or higher was used to indicate clinically significant anxiety in university students [31]. The STAI-6 is an extensively utilized psychometric questionnaire, which has been culturally adapted and validated for Greek populations [32]. In our current sample, the STAI-6 exhibited sufficient internal consistency (Cronbach’s α = 0.81).

#### 2.3.4. MD Adherence Assessment

Adherence to MD among university students was evaluated using the KIDMED questionnaire, a commonly applied tool for assessing MD compliance in children, adolescents, and young adults [33,34]. This 16-item questionnaire is recognized for its strong reliability and validity in capturing dietary behaviors. Each item is answered with a simple “yes” or “no” and scored either +1 for favorable dietary habits or −1 for habits contrary to the MD. Specifically, 12 items reflect positive adherence (e.g., regular consumption of fruits, vegetables, fish, legumes, nuts, dairy, and use of olive oil), while four items assess negative behaviors (e.g., frequent consumption of fast food, baked goods, sweets, or skipping breakfast). The total score ranges from 0 to 12, with scores classified as follows: ≥8 indicating good adherence, 4–7 moderate adherence, and ≤3 poor adherence [33,34]. All the above data were collected during face-to-face interviews with qualified personnel. In our sample, the KIDMED index exhibited satisfactory internal consistency (Cronbach’s α = 0.86).

### 2.4. Statistical Analysis

Continuous variables following normal distribution were processed by Student’s *t*-test, whereas normality was evaluated by applying the Kolmogorov–Smirnov test. Categorical variables were explored with the use of the chi-square (χ^2^) test. Quantitative data are presented as mean values ± standard deviation (SD) for normally distributed variables, whereas qualitative data are reported as absolute numbers and corresponding percentages. Multivariate binary logistic regression analysis was used to assess whether university students’ MD adherence could independently be associated with sociodemographic, anthropometric, and lifestyle characteristics, including depression and anxiety, by adjusting for potential confounders. All the variables, except MD adherence, were taken into consideration as potential confounding factors. Results are presented with odds ratios (OR), 95% confidence intervals (CI), and *p*-values. A *p*-value below 0.05 was defined as statistically significant. The Statistica 10.0 software, Europe, was used for the statistical analysis (In-former Technologies, Inc., Hamburg, Germany).

## 3. Results

Table 1 presents the descriptive characteristics of the study population. A total of 5191 university students participated in the study, with a mean age of 21.3 ± 2.4 years. Of these, 52.0% were female, and 82.0% held Greek nationality. In terms of geographic distribution, 55.3% resided in urban areas, while 44.7% lived in rural regions. Socioeconomic status varied, with 40.8% of participants reporting low income, 37.2% medium income, and 22.2% high income. Regarding living arrangements, 56.2% lived with their families, whereas 43.8% lived alone. Additionally, 38.0% of students reported that their parents were divorced, and 39.0% were identified as regular smokers.

Additionally, 54.9% of the participating university students were enrolled in biomedical fields such as medicine, nursing, pharmacy, or nutrition. Regarding academic performance, 42.9% achieved good grades, 38.6% very good, and 18.5% excellent grades. Employment was reported by 36.1% of the students. In terms of weight status, 18.9% were classified as overweight and 8.4% as obese.

Regarding the lifestyle characteristics of the study population, 46.7% of the university students reported low levels of physical activity, 33.8% had moderate activity levels, and only 19.5% engaged in high physical activity. In terms of mental health, 33.9% of the participants exhibited depressive symptoms, while 34.9% showed signs of anxiety. Concerning dietary habits, 47.3% demonstrated low adherence to the MD, 34.7% had moderate adherence, and just 18.0% displayed high adherence.

### 3.1. Associations of MD Compliance with Sociodemographic, Anthropometric, and Lifestyle Factors

Cross-tabulation analysis revealed that female university students exhibited significantly higher adherence to the MD than their male counterparts (Table 2, *p* ˂ 0.001). Students residing in rural areas had greater MD adherence compared to those living in urban settings (Table 2, *p* < 0.0001). Similarly, those from families with high economic status demonstrated higher MD adherence than students from low-income households (Table 2, *p* ˂ 0.001). Greater adherence to the MD was also more common among students who lived with their families and among those whose parents were not divorced (Table 2, *p* < 0.0001). Finally, regular smoking was significantly more prevalent among students with low MD adherence compared to those with higher MD adherence levels (Table 2, *p* < 0.0001).

University students enrolled in biomedical sciences and those with excellent academic performance demonstrated significantly higher adherence to the MD (Table 2, *p* ˂ 0.001 and *p* ˂ 0.001, respectively). Non-employed students were also moderately more likely to have higher MD adherence compared to their employed peers (Table 2, *p* ˂ 0.01). Additionally, greater MD adherence was associated with higher levels of physical activity (*p* ˂ 0.001). Students classified as overweight or obese had notably lower adherence to the MD compared to those with normal weight (Table 2, *p* < 0.0001). Finally, lower MD adherence was significantly more common among students exhibiting symptoms of depression and anxiety (Table 2, *p* < 0.0001).

### 3.2. Multivariate Binary Logistic Regression Analysis for MD Compliance of the Study Population

Multivariate logistic regression analysis demonstrated that MD compliance was independently related to various factors such as gender, area of residence, living status, smoking habits, study domain, academic performance, physical activity, BMI, and the presence of depression or anxiety symptomatology (Table 3, *p* < 0.005). In the logistic regression in Table 3, the reference level for each variable is the first category mentioned (in parentheses), against which the second category was compared. An odds ratio (OR) equal to 1 means no difference, an OR above 1 means higher odds, and an OR below 1 means lower odds. In this respect, we found that female students had a 27% greater incidence of higher MD adherence compared to male students (Table 3, *p* ˂ 0.01). Students residing in rural areas were 13% more likely to have higher MD adherence than those living in urban areas (Table 3, *p* ˂ 0.01). Additionally, students living with family had 22% higher odds of greater MD adherence compared to those living alone (Table 3, *p* ˂ 0.01). Nonsmokers were also 25% more likely to demonstrate high adherence to the MD than smokers (Table 3, *p* ˂ 0.01).

University students pursuing biomedical sciences exhibited a 33% higher incidence of following MD at higher levels compared to those engaged in other sciences (Table 3, *p* ˂ 0.01). Students with very good or excellent academic performance exhibited a 17% higher frequency of following high MD compliance than those with good academic performance (Table 3, *p* ˂ 0.01). University students with higher levels of physical activity showed an 84% higher probability of demonstrating high MD adherence compared to those with lower physical activity levels (Table 3, *p* ˂ 0.001).

University students with low MD adherence had a more than two-fold prevalence of developing overweight or obesity (Table 3, *p* ˂ 0.001). Moreover, university students with low MD adherence exhibited a more than two-fold incidence of presenting symptoms of depression or anxiety (Table 3, *p* ˂ 0.001 and *p* ˂ 0.001, respectively).

## 4. Discussion

This study aimed to investigate the role of MD adherence on anxiety and depression in university students. According to our results, low adherence to the MD is independently associated with a significantly higher prevalence of depression, anxiety, and overweight/obesity.

***Associations of MD adherence with mental health***

These findings are aligned with a growing body of literature emphasizing the mental health benefits of MD adherence in academic settings [35,36,37,38,39]. For instance, nursing students with high MD adherence reported fewer depressive symptoms, reinforcing the influence of dietary patterns on young adults’ mental well-being [37]. Similarly, Trigueros et al. (2020) found that emotional factors like academic stress and exam-related anxiety negatively predicted MD adherence in university students [39], highlighting a bidirectional relationship between diet and mental health. Further supporting this connection, Morales et al. (2023) observed that students with moderate to high adherence to the MD had 36% lower odds of depressive symptoms [38]. Likewise, Chacón-Cuberos et al. (2019) reported that students with low MD adherence experienced higher stress [36]. Another cross-sectional study on 200 university students in Lebanon showed a significant inverse association between MD adherence and anxiety [40]. Similarly, a cross-sectional study on 558 Spanish university students also found that higher MD adherence was associated with lower levels of anxiety [41].

Findings from non-student populations further support the protective impact of the MD against mental disorders [42,43,44,45,46]. Jiménez-López et al. (2024) found a significant association between MD and depressive symptoms in adolescents [47]. On the other hand, a randomized controlled trial (RCT) observed no significant improvements in depression or anxiety scores immediately following an MD-based intervention [48]. Interestingly, after adjusting for baseline confounding factors, improvements in mental health outcomes did emerge, suggesting that adequate dietary compliance may be essential for observing benefits [48]. Variability in study designs, dietary assessment tools, and mental health measurement methods may account for these inconsistencies across the literature.

***Potential mechanisms of MD against mental disorders***

Diet likely plays an important role in mental health [49], as specific components of the MD, such as nuts, olive oil, fish, fruit and vegetables, and legumes, are rich in antioxidants, polyphenols, phytochemicals, and B-complex vitamins—particularly vitamins B12 and B9. These vitamins are essential for the synthesis of neurotransmitters like dopamine and serotonin [50]. Polyphenols (e.g., curcumin and walnut flavanols) have demonstrated antidepressant effects [17]. Additionally, chronic inflammation has been implicated in mental illness, with biomarkers such as interleukin-6 (IL-6), tumor necrosis factor-alpha (TNF-α), and C-reactive protein (CRP) being associated with depressive symptoms [51].

MD can exert its protective effects via several biological pathways, including the regulation of glucocorticoid activity [52], enhancement of neurogenesis [53], reduction in oxidative stress and inflammatory markers [54], and epigenetic regulation [55], as well as through modulation of gut microbiota [56]. A balanced gut microbiome (eubiosis) supports mental health by promoting the production of short-chain fatty acids and regulating the gut–brain axis [57]. Conversely, dysbiosis—an imbalance in gut microbiota—has been linked to neuroinflammation and mood disorders [58]. In this respect, our findings enhance the currently available evidence that dietary patterns such as MD can influence inflammatory processes, neurochemical pathways, or gut microbiota, ultimately affecting mood and mental health. In this regard, a cross-sectional survey showed that higher dietary inflammatory index levels were associated with increased risk of depressive symptoms and anxiety and lower probability of well-being [59]. A recent meta-analysis including 17 studies with a total of 157,409 participants also showed that long-term anti-inflammatory eating patterns may prevent depression and anxiety, whereas pro-inflammatory eating patterns may promote these conditions [60].

***MD, glycemic index, and ultra-processed foods intake***

The MD’s low glycemic index may contribute to its psychological benefits. Diets with a low glycemic load help maintain stable blood glucose levels and are associated with lower insulin resistance [61]. Since insulin resistance is linked to cognitive impairments and depressive symptoms [62], this may represent another pathway through which the MD could support mental health. Furthermore, stable glucose levels help prevent mood swings, fatigue, and irritability [63], which are common in high glycemic index diets. Finally, the MD is characterized by a low intake of ultra-processed foods [64], which have been strongly associated with depression, anxiety, and psychological distress [65]. These foods tend to be energy-dense but nutrient-poor, and their high content of additives, trans fats, and added sugars may exacerbate inflammation and dysregulate brain function [66].

***Impact of MD adherence on overweight and obesity***

Our study found that students with lower MD adherence were more than twice as likely to be overweight or obese, even after adjusting for confounding variables. However, the evidence regarding the relationship between MD adherence and excess weight in academic settings remains limited and contradictory [35,37]. Some studies conducted among university students have reported no significant associations [35,37], while others have found a clear relationship [63]. For example, among nursing students, MD adherence did not differ significantly across BMI categories, with no notable differences between normal-weight and overweight/obese individuals [37]. In contrast, in another study, overweight students showed lower MD adherence, yet it was significantly higher compared with those with a normal weight (15.5% vs. 8.5%), suggesting that students with poor dietary habits were more likely to be overweight [67].

In addition, studies conducted in non-student populations have shown more varied results, with some reporting significant associations between MD adherence and BMI [42,47], whereas others found no relationship [46,49]. For instance, in overweight/obese women, greater MD adherence was associated with lower BMI [42]. Similarly, Romaguera et al. (2009), in a large-scale study involving 351,730 individuals from nine European countries, reported that higher MD adherence was significantly associated with a lower prevalence of abdominal obesity in both men and women [68]. In an RCT, participants in the MD group had significantly lower post-intervention weight and BMI compared to the control group [48]. Furthermore, Sadeghi et al. (2021) observed a trend of decreasing BMI and prevalence of overweight/obesity with higher MD adherence [46]. On the other hand, Leone et al. (2022) found no significant association between MD adherence and metabolic health status among obese individuals [69]. The inconsistencies across studies regarding the association between MD adherence and BMI may be attributed to several factors. Study design, variability in assessment tools, and self-reported methods are among these factors.

An additional possible explanation for the observed association between MD adherence and lower BMI is that individuals who follow this dietary pattern are generally more health-conscious and tend to engage in other healthy lifestyle behaviors. For example, they may also be more physically active [69], avoid smoking, and get adequate sleep. These combined behaviors contribute to better weight management. Additionally, the MD itself is rich in fiber, healthy fats, and low-glycemic-index foods, which can enhance satiety, reduce overeating, and support metabolic regulation.

***MD adherence and physical activity***

We also observed that students with low MD adherence were more likely to report lower levels of physical activity. This finding is supported by previous research suggesting a positive association between MD adherence and physical fitness [35,45,69]. A meta-analysis demonstrated that individuals with higher MD adherence tended to have better physical fitness levels [70]. Similarly, Alfaro-González et al. (2025) reported that, among university students, greater MD adherence was associated with increased lean mass and improvements in the muscle skeletal index [35]. Recently, Houminer-Klepar and Dopelt (2025) also found that higher levels of exercise may reinforce healthier eating patterns in adults [45]. This association may be partly explained by the fact that, especially among young people, concerns about body image and the desire for physical attractiveness often motivate the adoption of healthier behaviors, including regular exercise and better dietary choices. As a result, students who are more physically active may also be more inclined to follow balanced dietary patterns such as the MD.

***MD adherence and smoking status***

In our study, students with low MD adherence were more often smokers, suggesting a link between unhealthy dietary patterns and other adverse lifestyle behaviors. This finding aligns with previous research [37,71]. Ibáñez-del Valle et al. (2023) reported that nursing students who smoked were more likely to have lower MD adherence [37]. Similarly, a large prospective cohort found that current smokers with low MD adherence had a significantly higher risk of all-cause and cancer-related mortality, emphasizing the harmful synergy between tobacco use and poor dietary quality [71]. Jiménez-López et al. (2024) also observed a trend indicating lower smoking rates among individuals with higher MD adherence, further supporting the notion that healthy behaviors often cluster together [47].

***MD adherence and students’ gender***

We also found that low MD adherence was more common among male students. While some previous research found no significant association between MD adherence and gender [37,38,47], other studies support our finding [71,72]. For instance, Baydemir et al. (2018) reported that female university students showed better MD adherence than males [72]. The existing discrepancies may be partly explained by biological and behavioral differences between sexes. Males and females have distinct nutritional requirements, which can influence dietary preferences and habits [73].

***MD adherence and students’ living status***

Our results also showed that students living alone were more likely to have low MD adherence. This finding is consistent with previous studies reporting that students who live with their families or in dormitories tend to follow healthier eating patterns [63,72,73,74,75,76]. Baydemir et al. (2018) observed significantly higher KIDMED scores among students living with family [72]. Similarly, El-Kassas and Ziade (2016) and Pelletier and Laska (2013) found that students living in dormitories or independently were more likely to adopt unhealthy dietary habits, often due to a lack of structure or cooking skills [75,76]. Durá Travé and Castroviejo Gandarias (2011) also found markedly higher MD adherence among students living at home compared to those in residences or apartments [67].

***MD adherence and students’ academic performance***

We also observed that students with low MD adherence were more likely to have lower academic performance. Although limited, the existing literature supports this association [77,78]. In a Spanish cohort, Alfaro-González et al. (2024) found that students with higher MD adherence achieved significantly better academic outcomes, particularly when their diets were rich in olive oil, fruits, legumes, vegetables, and fish [77]. These foods include high levels of omega-3 polyunsaturated fatty acids, which have been shown to support cognitive function and may contribute to improved academic performance [79].

***MD adherence and future perspectives***

The above findings carry important implications for both clinical practice and public health policy. With the growing recognition of lifestyle medicine as a valuable complement to traditional psychiatric care, healthcare professionals should consider incorporating dietary assessment and counseling into mental health interventions. Promoting adherence to the MD may serve as a low-risk, cost-effective strategy to support psychological well-being, particularly in individuals experiencing mild-to-moderate symptoms of mental health disorders.

At the public health level, our results underscore the need for educational initiatives that emphasize the mental and physical health benefits of balanced dietary patterns. Programs promoting whole-food-based nutrition should be integrated into school curricula, university settings, and workplace wellness programs. Given the tendency for health-related behaviors to cluster, interventions that combine dietary education with the promotion of physical activity, smoking prevention, and stress management may offer synergistic benefits. Tailored strategies targeting university populations, who face unique lifestyle challenges, could be particularly impactful in shaping long-term habits during a formative life stage.

***Strengths and Limitations***

To our knowledge, the present study is one of the first to investigate the association between MD adherence and both anxiety and depression symptoms in Greek university students. Moreover, it considers multiple lifestyle factors, including smoking, physical activity, and academic performance, providing a more comprehensive understanding of how MD adherence fits within a broader health profile in the population. In addition, not all data were self-reported, as they were collected by face-to-face interviews, which may minimize recall bias or social desirability bias, particularly in areas such as diet, mental health, or body weight. Finally, it includes a relatively large and diverse sample of university students, enhancing the statistical power and reliability of the results.

This study has certain limitations. First, the cross-sectional design prevents any conclusions about causality between MD adherence and mental health outcomes. Given the bidirectional relationship between diet and mental health, this is a critical limitation. Second, even if all data were not self-reported, recall bias or social desirability bias may be retained. Third, the sample was limited to students from Greece, which may affect the generalizability of the findings to broader populations. Fourthly, the enrollment in the study took a long period of time, from March 2021 to October 2024, due to the fact that we wanted to include a large study population for face-to-face interviews, which increased the period of study enrollment. Even if this is a strength of the study, it is also a limitation, since the COVID-19 impact and other unmeasured potential environmental effects have been shown to increase the prevalence of psychiatric disorders, such as depression and anxiety, and this issue was not taken into consideration in the present study [80]. Fifthly, even if KIDMED has been validated for young adults (university or college students) of certain nationalities, there is so far no study examining the reliability of the Greek version of KIDMED. This undermines confidence in dietary adherence measurement. It also should be noted that our mental health outcomes (depression and anxiety) are based on self-reported symptom measures rather than clinical diagnoses. Thus, depression and anxiety measured with BD-II and STAI could be influenced by multiple factors beyond diet, including situational or acute stressors. Sleep disorders may also be associated with depression and anxiety, and vice versa [81]. However, we did not have any data concerning the sleep quality of our study population, which is another limitation of our study.

Moreover, in the multivariate analysis of our study, some ORs were close to 1 and should be interpreted cautiously, since the practical implications of such associations are less clear-cut. Especially, we should acknowledge that while the findings are statistically robust, the clinical or practical significance of certain associations may be limited. In addition, reverse causality between MD adherence and depression and anxiety could not be excluded due to the cross-sectional design of the study. Even if we used a large number of confounding factors, the presence of other potential confounders cannot be excluded. Thus, we must acknowledge possible residual confounding, since additional lifestyle factors such as alcohol use, stress, and sleep quality may be related to MD adherence. Lastly, given that data collection spanned from March 2021 to October 2024, the study inevitably overlaps with the COVID-19 pandemic. Hence, pandemic-related disruptions, including changes in university attendance, increased social isolation, and altered dietary behaviors, may have influenced both mental health and lifestyle patterns [82,83]. Thus, we aim to assess in the future any potential influence of COVID-19 confinement on psychological, behavioral, and physical activity implications on the lifestyle of university students.

## 5. Conclusions

The present study provided evidence that low MD adherence was significantly associated with increased prevalence of depression, anxiety, and excess body weight among university Greek students. These findings highlight the clustering of unhealthy lifestyle behaviors and emphasize the need for holistic strategies to promote mental and physical well-being among young adults. Targeted university-based interventions, focusing on nutrition education and healthy lifestyle promotion, can serve as cost-effective approaches to mitigate mental health risks and support healthier habits during this academic period. Based on the limitations of the current study, future longitudinal and interventional studies are highly warranted to clarify the directionality and causal nature of the underlying associations. Moreover, due to the limitations of the present study, future studies should apply subgroup or sensitivity analyses, such as stratification by gender, socioeconomic status, or academic discipline, to assess whether the associations between MD adherence and mental health outcomes vary across specific demographic or lifestyle factors, thereby offering more nuanced insights. In addition, future studies should perform additional analysis by year to rule out any confounding effects of COVID-19 confinement on the psychological and behavioral aspects of the lifestyle of university students.

## Figures and Tables

**Table 1:** Descriptive statistics of the enrolled university students.

Characteristics (*n* = 5191)	Descriptive Statistics
**Age (mean ± SD; years)**	21.3 ± 2.4
**Gender (*n*, %)**	
Male	2490 (48.0%)
Female	2701 (52.0%)
**Nationality (*n*, %)**	
Greek	4255 (82.0%)
Other	936 (18.0%)
**Type of residence (*n*, %)**	
Urban	2869 (55.3%)
Rural	2322 (44.7%)
**Family economic status**	
Low	2110 (40.8%)
Medium	1933 (37.2%)
High	1148 (22.1%)
**Living status (*n*, %)**	
Living with family	2919 (56.2%)
Living alone	2272 (43.8%)
**Parents marital status**	
Not divorced	3218 (62.0%)
Divorced	1973 (38.0%)
**Smoking status**	
No smokers	3157 (61.0%)
Smokers	2034 (39.0%)
**Type of Studies**	
Biomedical studies	2849 (54.9%)
Other studies	2342 (45.1%)
**Academic performance**	
Good	2225 (42.9%)
Very good	2005 (38.6%)
Excellent	961 (18.5%)
**Employment status**	
Employee	1872 (36.1%)
Not employee	3319 (63.9%)
**Physical activity (*n*, %)**	
Low	2427 (46.7%)
Medium	1754 (33.8%)
High	1010 (19.5%)
**BMI ^1^ (*n*, %)**	
Normal weight	3772 (72.7%)
Overweight	980 (18.9%)
Obese	439 (8.4%)
**Depression (*n*, %)**	
No	3433 (66.1%)
Yes	1758 (33.9%)
**Anxiety (*n*, %)**	
No	3380 (65.1%)
Yes	1811 (34.9%)
**Mediterranean Diet Adherence (*n*, %)**	
Low	2452 (47.3%)
Moderate	1803 (34.7%)
High	936 (18.0%)

^1^ BMI: body mass index.

**Table 2:** Associations of MD adherence with sociodemographic, anthropometric, and lifestyle factors of the enrolled university students.

Characteristics (*n* = 5191)	MD ^1^ Adherence			
	Low 2452 (47.3%)	Moderate 1803 (34.7%)	High 936 (18.0%)	*p*-Value ^2^
**Age (mean ± SD; years)**	21.5 ± 2.3	21.3 ± 2.4	21.1 ± 2.53	*p* = 0.174
**Gender (*n*, %)**				*p* ˂ 0.001
Male	1254 (51.1%)	874 (48.5%)	362 (38.7%)	
Female	1198 (48.9%)	929 (51.5%)	574 (61.3%)	
**Nationality (*n*, %)**				*p* = 0.184
Greek	2028 (82.7%)	1547 (85.8%)	680 (72.7%)	
Other	424 (17.3%)	256 (14.2%)	256 (27.3%)	
**Type of residence (*n*, %)**				*p* ˂ 0.0001
Urban	1622 (66.1%)	1035 (57.4%)	212 (22.7%)	
Rural	830 (33.9%)	768 (42.6%)	724 (77.3%)	
**Family economic status**				*p* ˂ 0.001
Low	1300 (53.0%)	492 (27.3%)	318 (34.0%)	
Medium	666 (27.2%)	899 (49.9%)	368 (39.3%)	
High	486 (19.8%)	412 (22.8%)	250 (26.7%)	
**Living status (*n*, %)**				*p* ˂ 0.0001
Living with family	892 (36.4%)	1241 (68.8%)	786 (84.0%)	
Living alone	1560 (63.6%)	562 (31.2%)	150 (16.0%)	
**Parents marital status**				*p* ˂ 0.0001
Not divorced	1261 (51.4%)	1211 (67.2%)	746 (79.7%)	
Divorced	1191 (48.6%)	592 (32.8%)	190 (20.3%)	
**Smoking status**				*p* ˂ 0.0001
No smokers	1154 (47.1%)	1279 (70.9%)	724 (77.4%)	
Regular smokers	1298 (52.9%)	524 (29.1%)	212 (22.6%)	
**Type of Studies**				*p* ˂ 0.001
Biomedical studies	1156 (47.1%)	1062 (58.9%)	629 (67.2%)	
Other studies	1296 (52.9%)	741 (41.1%)	307 (32.8%)	
**Academic performance**				*p* ˂ 0.001
Good	1490 (60.8%)	452 (25.1%)	283 (30.2%)	
Very good	792 (32.3%)	1014 (56.2%)	199 (21.3%)	
Excellent	170 (6.9%)	337 (18.7%)	454 (48.5%)	
**Employment status**				*p* ˂ 0.01
Not employee	1448 (59.1%)	1241 (68.8%)	630 (67.3%)	
Employee	1004 (40.9%)	562 (31.2%)	306 (32.7%)	
**Physical activity (*n*, %)**				*p* ˂ 0.001
Low	1939 (79.1%)	320 (17.7%)	168 (17.9%)	
Medium	359 (14.6%)	1278 (70.9%)	117 (12.5%)	
High	154 (6.3%)	205 (11.4%)	651 (69.6%)	
**BMI ^3^ (*n*, %)**				*p* ˂ 0.0001
Normal weight	1859 (75.8%)	1325 (73.5%)	588 (62.8%)	
Overweight	413 (16.8%)	370 (20.5%)	197 (21.1%)	
Obese	180 (7.4%)	108 (6.0%)	151 (16.1%)	
**Depression (*n*, %)**				*p* ˂ 0.0001
No	1336 (54.5%)	1317 (73.0%)	780 (83.3%)	
Yes	1116 (45.5%)	486 (47.0%)	156 (16.7%)	
**Anxiety (*n*, %)**				*p* ˂ 0.0001
No	1293 (52.7%)	1264 (70.1%)	823 (12.1%)	
Yes	1159 (47.3%)	539 (29.9%)	113 (87.9%)	

^1^ MD: Mediterranean diet. ^2^ Student’s *t*-test was used for age. Chi-square test was used for all the other variables. ^3^ BMI: body mass index.

**Table 3:** Multivariate analysis of MD adherence in the study population.

Variables ^1^	MD ^2^ Adherence (Low vs. Moderate and High)	
	OR ^3^ (95% CI ^4^)	*p*-Value
**Age (Over/Below mean value)**	1.27 (0.23–2.11)	*p* = 0.319
**Gender (Male/Female)**	0.73 (0.58–0.97)	*p* ˂ 0.01
**Nationality (Greek/Other)**	1.09 (0.38–1.85)	*p* = 0.453
**Type of residence (Urban/Rural)**	0.87 (0.64–1.08)	*p* ˂ 0.01
**Family economic status (Low/Medium and High)**	0.81 (0.35–1.32)	*p* = 0.189
**Living status (** **Living alone/Living with family)**	0.78 (0.61–0.92)	*p* ˂ 0.01
**Parents’ marital status (Divorced/Not divorced)**	0.92 (0.38–1.69)	*p* = 0.210
**Smoking status (Regular smokers/No smokers)**	0.75 (0.57–0.99)	*p* ˂ 0.01
**Type of Studies (Biomedical studies/Other studies)**	0.67 (0.49–0.86)	*p* = 0.021
**Academic performance (Good/Very good and Excellent)**	0.83 (0.65–1.02)	*p* ˂ 0.01
**Employment status (Not employee/Employee)**	1.22 (0.67–1.89)	*p* = 0.349
**Physical activity (Medium and High/Low)**	1.84 (1.62–2.05)	*p* ˂ 0.001
**BMI ^5^ (overweight and obese/normal weight)**	2.45 (2.18–2.69)	*p* ˂ 0.001
**Depression (Yes/No)**	2.12 (1.89–2.44)	*p* ˂ 0.001
**Anxiety (Yes/No)**	2.27 (2.01–2.52)	*p* ˂ 0.001

^1^ All the variables were used as confounding factors. ^2^ MD: Mediterranean diet. ^3^ OR: odds ratio. ^4^ CI: confidence interval. ^5^ BMI: body mass index.

## Data Availability

The data of the study are available upon request to the corresponding authors.

## References

[1] Jalali A., Ziapour A., Karimi Z., Rezaei M., Emami B., Kalhori R.P., Khosravi F., Sameni J.S., Kazeminia M. (2024). Global Prevalence of Depression, Anxiety, and Stress in the Elderly Population: A Systematic Review and Meta-Analysis. BMC Geriatr..

[2] Seighali N., Abdollahi A., Shafiee A., Amini M.J., Teymouri Athar M.M., Safari O., Faghfouri P., Eskandari A., Rostaii O., Salehi A.H. (2024). The global prevalence of depression, anxiety, and sleep disorder among patients coping with Post COVID-19 syndrome (long COVID): A systematic review and meta-analysis. BMC Psychiatry.

[3] Arias D., Saxena S., Verguet S. (2022). Quantifying the Global Burden of Mental Disorders and Their Economic Value. eClinicalMedicine.

[4] Grajek M., Krupa-Kotara K., Białek-Dratwa A., Sobczyk K., Grot M., Kowalski O., Staśkiewicz W. (2022). Nutrition and Mental Health: A Review of Current Knowledge about the Impact of Diet on Mental Health. Front. Nutr..

[5] Cater R.J., Chua G.L., Erramilli S.K., Keener J.E., Choy B.C., Tokarz P., Chin C.F., Quek D.Q.Y., Kloss B., Pepe J.G. (2021). Structural Basis of Omega-3 Fatty Acid Transport across the Blood-Brain Barrier. Nature.

[6] Fernández M.J.F., Valero-Cases E., Rincon-Frutos L. (2019). Food Components with the Potential to Be Used in the Therapeutic Approach of Mental Diseases. Curr. Pharm. Biotechnol..

[7] Mulati A., Ma S., Zhang H., Ren B., Zhao B., Wang L., Liu X., Zhao T., Kamanova S., Sair A.T. (2020). Sea-Buckthorn Flavonoids Alleviate High-Fat and High-Fructose Diet-Induced Cognitive Impairment by Inhibiting Insulin Resistance and Neuroinflammation. J. Agric. Food Chem..

[8] Park M., Choi J., Lee H.-J. (2020). Flavonoid-Rich Orange Juice Intake and Altered Gut Microbiome in Young Adults with Depressive Symptom: A Randomized Controlled Study. Nutrients.

[9] Dakanalis A., Voulgaridou G., Alexatou O., Papadopoulou S.K., Jacovides C., Pritsa A., Chrysafi M., Papacosta E., Kapetanou M.G., Tsourouflis G. (2024). Overweight and Obesity Is Associated with Higher Risk of Perceived Stress and Poor Sleep Quality in Young Adults. Medicina.

[10] Beiter R., Nash R., McCrady M., Rhoades D., Linscomb M., Clarahan M., Sammut S. (2015). The Prevalence and Correlates of Depression, Anxiety, and Stress in a Sample of College Students. J. Affect. Disord..

[11] Bayram N., Bilgel N. (2008). The Prevalence and Socio-Demographic Correlations of Depression, Anxiety and Stress among a Group of University Students. Soc. Psychiat Epidemiol..

[12] Pedrelli P., Nyer M., Yeung A., Zulauf C., Wilens T. (2015). College Students: Mental Health Problems and Treatment Considerations. Acad. Psychiatry.

[13] da Costa Pelonha R.N., Jomori M.M., Maciel T.G., Rocha J.A.D., Passos T.S., Maciel B.L.L. (2023). Low Cooking Skills Are Associated with Overweight and Obesity in Undergraduates. Nutrients.

[14] Gesteiro E., García-Carro A., Aparicio-Ugarriza R., González-Gross M. (2022). Eating out of Home: Influence on Nutrition, Health, and Policies: A Scoping Review. Nutrients.

[15] Magni O., Detopoulou P., Fappa E., Perrea A., Levidi D., Dedes V., Tzoutzou M., Gioxari A., Panoutsopoulos G. (2024). Eating Attitudes, Stress, Anxiety, and Depression in Dietetic Students and Association with Body Mass Index and Body Fat Percent: A Cross-Sectional Study. Diseases.

[16] Dominguez L.J., Di Bella G., Veronese N., Barbagallo M. (2021). Impact of Mediterranean Diet on Chronic Non-Communicable Diseases and Longevity. Nutrients.

[17] Bayes J., Schloss J., Sibbritt D. (2020). Effects of Polyphenols in a Mediterranean Diet on Symptoms of Depression: A Systematic Literature Review. Adv. Nutr..

[18] Trichopoulou A., Lagiou P. (1997). Healthy Traditional Mediterranean Diet: An Expression of Culture, History, and Lifestyle. Nutr. Rev..

[19] Deacon G., Kettle C., Hayes D., Dennis C., Tucci J. (2017). Omega 3 Polyunsaturated Fatty Acids and the Treatment of Depression. Crit. Rev. Food Sci. Nutr..

[20] Liao Y., Xie B., Zhang H., He Q., Guo L., Subramanieapillai M., Fan B., Lu C., McIntyre R.S. (2019). Efficacy of Omega-3 PUFAs in Depression: A Meta-Analysis. Transl. Psychiatry.

[21] Frangou S., Lewis M., Wollard J., Simmons A. (2007). Preliminary in Vivo Evidence of Increased N-Acetyl-Aspartate Following Eicosapentanoic Acid Treatment in Patients with Bipolar Disorder. J. Psychopharmacol..

[22] Rao M.S., Hattiangady B., Shetty A.K. (2006). Fetal Hippocampal CA3 Cell Grafts Enriched with FGF-2 and BDNF Exhibit Robust Long-Term Survival and Integration and Suppress Aberrant Mossy Fiber Sprouting in the Injured Middle-Aged Hippocampus. Neurobiol. Dis..

[23] Su K.-P., Lai H.-C., Yang H.-T., Su W.-P., Peng C.-Y., Chang J.P.-C., Chang H.-C., Pariante C.M. (2014). Omega-3 Fatty Acids in the Prevention of Interferon-Alpha-Induced Depression: Results from a Randomized, Controlled Trial. Biol. Psychiatry.

[24] Antonopoulou M., Mantzorou M., Serdari A., Bonotis K., Vasios G., Pavlidou E., Trifonos C., Vadikolias K., Petridis D., Giaginis C. (2020). Evaluating Mediterranean Diet Adherence in University Student Populations: Does This Dietary Pattern Affect Students’ Academic Performance and Mental Health?. Int. J. Health Plan. Manag..

[25] James W.P.T. (2008). WHO Recognition of the Global Obesity Epidemic. Int. J. Obes..

[26] World Health Organization (2006). Rapport Sur La Situation Dans Le Monde: 2006: Travailler Ensemble Pour La Santé.

[27] Craig C.L., Marshall A.L., Sjöström M., Bauman A.E., Booth M.L., Ainsworth B.E., Pratt M., Ekelund U., Yngve A., Sallis J.F. (2003). International Physical Activity Questionnaire: 12-Country Reliability and Validity. Med. Sci. Sports Exerc..

[28] Papathanasiou G., Georgoudis G., Papandreou M., Spyropoulos P., Georgakopoulos D., Kalfakakou V., Evangelou A. (2009). Reliability measures of the short International Physical Activity Questionnaire (IPAQ) in Greek young adults. Hell. J. Cardiol..

[29] Wang Y.-P., Gorenstein C. (2013). Psychometric Properties of the Beck Depression Inventory-II: A Comprehensive Review. Braz. J. Psychiatry.

[30] Fountoulakis K., Iacovides A., Kleanthous S., Samolis S., Kaprinis S.G., Sitzoglou K., Kaprinis S.G., Bech P. (2001). Reliability, validity and psychometric properties of the Greek translation of the Center for Epidemiological Studies-Depression (CES-D) Scale. BMC Psychiatry.

[31] Chlan L., Savik K., Weinert C. (2003). Development of a Shortened State Anxiety Scale from the Spielberger State-Trait Anxiety Inventory (STAI) for Patients Receiving Mechanical Ventilatory Support. J. Nurs. Meas..

[32] Psychountaki M., Zervas Y., Karteroliotis K., Spielberger C. (2003). Reliability and validity of the Greek version of the STAIC. Eur. J. Psychol. Assess..

[33] Štefan L., Prosoli R., Juranko D., Čule M., Milinović I., Novak D., Sporiš G. (2017). The Reliability of the Mediterranean Diet Quality Index (KIDMED) Questionnaire. Nutrients.

[34] Atencio-Osorio M.A., Carrillo-Arango H.A., Correa-Rodríguez M., Ochoa-Muñoz A.F., Ramírez-Vélez R. (2020). Adherence to the Mediterranean Diet in College Students: Evaluation of Psychometric Properties of the KIDMED Questionnaire. Nutrients.

[35] Alfaro-González S., Garrido-Miguel M., Pascual-Morena C., Pozuelo-Carrascosa D.P., Fernández-Rodríguez R., Martínez-Hortelano J.A., Mesas A.E., Martínez-Vizcaíno V. (2025). The Association Between Adherence to the Mediterranean Diet and Depression and Anxiety Symptoms in University Students: The Mediating Role of Lean Mass and the Muscle Strength Index. Nutrients.

[36] Chacón-Cuberos R., Zurita-Ortega F., Olmedo-Moreno E.M., Castro-Sánchez M. (2019). Relationship between Academic Stress, Physical Activity and Diet in University Students of Education. Behav. Sci..

[37] Ibáñez-del Valle V., Navarro-Martínez R., Cauli O. (2023). Association between Depressive Symptoms and Adherence to the Mediterranean Diet in Nursing Students. Nutrients.

[38] Morales G., Balboa-Castillo T., Fernández-Rodríguez R., Garrido-Miguel M., Guidoni C.M., Sirtoli R., Mesas A.E., Rodrigues R. (2023). Adherence to the Mediterranean Diet and Depression, Anxiety, and Stress Symptoms in Chilean University Students: A Cross-Sectional Study. Cad. Saúde Pública.

[39] Trigueros R., Padilla A.M., Aguilar-Parra J.M., Rocamora P., Morales-Gázquez M.J., López-Liria R. (2020). The Influence of Emotional Intelligence on Resilience, Test Anxiety, Academic Stress and the Mediterranean Diet. A Study with University Students. Int. J. Environ. Res. Public Health.

[40] El Mikkawi H., El Khoury C., Rizk R. (2024). Adherence to the Mediterranean diet and mental health among university students in Lebanon. Appl. Food Res..

[41] Melguizo-Ibáñez E., González-Valero G., Badicu G., Yagin F.H., Alonso-Vargas J.M., Ardigò L.P., Puertas-Molero P. (2023). Mediterranean diet adherence on self-concept and anxiety as a function of weekly physical activity: An explanatory model in higher education. Front. Nutr..

[42] Zamani B., Zeinalabedini M., Nasli Esfahani E., Azadbakht L. (2023). Can Following Paleolithic and Mediterranean Diets Reduce the Risk of Stress, Anxiety, and Depression: A Cross-Sectional Study on Iranian Women. J. Nutr. Metab..

[43] Shiraseb F., Mirzababaei A., Daneshzad E., Khosravinia D., Clark C.C.T., Mirzaei K. (2023). The Association of Dietary Approaches to Stop Hypertension (DASH) and Mediterranean Diet with Mental Health, Sleep Quality and Chronotype in Women with Overweight and Obesity: A Cross-Sectional Study. Eat. Weight Disord..

[44] Dinu M., Lotti S., Napoletano A., Corrao A., Pagliai G., Tristan Asensi M., Gianfredi V., Nucci D., Colombini B., Sofi F. (2022). Association between Psychological Disorders, Mediterranean Diet, and Chronotype in a Group of Italian Adults. Int. J. Environ. Res. Public Health.

[45] Houminer-Klepar N., Dopelt K. (2025). Associations Between Mediterranean Diet, Processed Food Consumption, and Symptoms of Anxiety and Depression: Cross-Sectional Study Among Israeli Adults. Foods.

[46] Sadeghi O., Keshteli A.H., Afshar H., Esmaillzadeh A., Adibi P. (2021). Adherence to Mediterranean Dietary Pattern Is Inversely Associated with Depression, Anxiety and Psychological Distress. Nutr. Neurosci..

[47] Jiménez-López E., Mesas A.E., Visier-Alfonso M.E., Pascual-Morena C., Martínez-Vizcaíno V., Herrera-Gutiérrez E., López-Gil J.F. (2024). Adherence to the Mediterranean Diet and Depressive, Anxiety, and Stress Symptoms in Spanish Adolescents: Results from the EHDLA Study. Eur. Child. Adolesc. Psychiatry.

[48] Radkhah N., Rasouli A., Majnouni A., Eskandari E., Parastouei K. (2023). The Effect of Mediterranean Diet Instructions on Depression, Anxiety, Stress, and Anthropometric Indices: A Randomized, Double-Blind, Controlled Clinical Trial. Prev. Med. Rep..

[49] Ventriglio A., Sancassiani F., Contu M.P., Latorre M., Di Salvatore M., Fornaro M., Bhugra D. (2020). Mediterranean Diet and Its Benefits on Health and Mental Health: A Literature Review. Clin. Pract. Epidemiol. Ment. Health.

[50] Orywal K., Socha K., Iwaniuk P., Kaczyński P., Farhan J.A., Zoń W., Łozowicka B., Perkowski M., Mroczko B. (2025). Vitamins in the Prevention and Support Therapy of Neurodegenerative Diseases. Int. J. Mol. Sci..

[51] Osimo E.F., Pillinger T., Rodriguez I.M., Khandaker G.M., Pariante C.M., Howes O.D. (2020). Inflammatory Markers in Depression: A Meta-Analysis of Mean Differences and Variability in 5166 Patients and 5083 Controls. Brain Behav. Immun..

[52] García-Prieto M.D., Tébar F.J., Nicolás F., Larqué E., Zamora S., Garaulet M. (2007). Cortisol Secretary Pattern and Glucocorticoid Feedback Sensitivity in Women from a Mediterranean Area: Relationship with Anthropometric Characteristics, Dietary Intake and Plasma Fatty Acid Profile. Clin. Endocrinol..

[53] Gómez-Pinilla F. (2008). Brain Foods: The Effects of Nutrients on Brain Function. Nat. Rev. Neurosci..

[54] Sureda A., Bibiloni M.D.M., Julibert A., Bouzas C., Argelich E., Llompart I., Pons A., Tur J.A. (2018). Adherence to the Mediterranean Diet and Inflammatory Markers. Nutrients.

[55] Kenanoglu S., Gokce N., Akalin H., Ergoren M.C., Beccari T., Bertelli M., Dundar M. (2022). Implication of the Mediterranean Diet on the Human Epigenome. J. Prev. Med. Hyg..

[56] Merra G., Noce A., Marrone G., Cintoni M., Tarsitano M.G., Capacci A., De Lorenzo A. (2020). Influence of Mediterranean Diet on Human Gut Microbiota. Nutrients.

[57] Silva Y.P., Bernardi A., Frozza R.L. (2020). The Role of Short-Chain Fatty Acids From Gut Microbiota in Gut-Brain Communication. Front. Endocrinol..

[58] Dinan T.G., Cryan J.F. (2017). Gut Instincts: Microbiota as a Key Regulator of Brain Development, Ageing and Neurodegeneration. J. Physiol..

[59] Phillips C.M., Shivappa N., Hébert J.R., Perry I.J. (2018). Dietary inflammatory index and mental health: A cross-sectional analysis of the relationship with depressive symptoms, anxiety and well-being in adults. Clin. Nutr..

[60] Li X., Chen M., Yao Z., Zhang T., Li Z. (2022). Dietary inflammatory potential and the incidence of depression and anxiety: A meta-analysis. J. Health Popul. Nutr..

[61] Haghighatdoost F., Azadbakht L., Keshteli A.H., Feinle-Bisset C., Daghaghzadeh H., Afshar H., Feizi A., Esmaillzadeh A., Adibi P. (2016). Glycemic Index, Glycemic Load, and Common Psychological Disorders. Am. J. Clin. Nutr..

[62] Hamer J.A., Testani D., Mansur R.B., Lee Y., Subramaniapillai M., McIntyre R.S. (2019). Brain Insulin Resistance: A Treatment Target for Cognitive Impairment and Anhedonia in Depression. Exp. Neurol..

[63] Breymeyer K.L., Lampe J.W., McGregor B.A., Neuhouser M.L. (2016). Subjective Mood and Energy Levels of Healthy Weight and Overweight/Obese Healthy Adults on High-and Low-Glycemic Load Experimental Diets. Appetite.

[64] Dinu M., Tristan Asensi M., Pagliai G., Lotti S., Martini D., Colombini B., Sofi F. (2022). Consumption of Ultra-Processed Foods Is Inversely Associated with Adherence to the Mediterranean Diet: A Cross-Sectional Study. Nutrients.

[65] Pagliai G., Dinu M., Madarena M.P., Bonaccio M., Iacoviello L., Sofi F. (2021). Consumption of Ultra-Processed Foods and Health Status: A Systematic Review and Meta-Analysis. Br. J. Nutr..

[66] Monteiro C.A., Cannon G., Levy R.B., Moubarac J.-C., Louzada M.L., Rauber F., Khandpur N., Cediel G., Neri D., Martinez-Steele E. (2019). Ultra-Processed Foods: What They Are and How to Identify Them. Public Health Nutr..

[67] Durá Travé T., Castroviejo Gandarias A. (2011). Adherence to a Mediterranean diet in a college population. Nutr. Hosp..

[68] Romaguera D., Norat T., Mouw T., May A.M., Bamia C., Slimani N., Travier N., Besson H., Luan J., Wareham N. (2009). Adherence to the Mediterranean Diet Is Associated with Lower Abdominal Adiposity in European Men and Women. J. Nutr..

[69] Leone A., De Amicis R., Battezzati A., Bertoli S. (2022). Adherence to the Mediterranean Diet and Risk of Metabolically Unhealthy Obesity in Women: A Cross-Sectional Study. Front. Nutr..

[70] Bizzozero-Peroni B., Brazo-Sayavera J., Martínez-Vizcaíno V., Fernández-Rodríguez R., López-Gil J.F., Díaz-Goñi V., Cavero-Redondo I., Mesas A.E. (2022). High Adherence to the Mediterranean Diet Is Associated with Higher Physical Fitness in Adults: A Systematic Review and Meta-Analysis. Adv. Nutr..

[71] Pardavila-Belio M.I., de la O V., Hershey M.S., Barbería-Latasa M., Toledo E., Martin-Moreno J.M., Martínez-González M.Á., Ruiz-Canela M. (2022). Joint Association of the Mediterranean Diet and Smoking with All-Cause Mortality in the Seguimiento Universidad de Navarra (SUN) Cohort. Nutrition.

[72] Baydemir C., Ozgur E.G., Balci S. (2018). Evaluation of Adherence to Mediterranean Diet in Medical Students at Kocaeli University, Turkey. J. Int. Med. Res..

[73] Li K.-K., Concepcion R.Y., Lee H., Cardinal B.J., Ebbeck V., Woekel E., Readdy R.T. (2012). An Examination of Sex Differences in Relation to the Eating Habits and Nutrient Intakes of University Students. J. Nutr. Educ. Behav..

[74] López Torres O., Fernández-Elías V.E. (2024). Training and Nutrition for Performance: Males, Females, and Gender Differences. Nutrients.

[75] El-Kassas G., Ziade F. (2016). Exploration of the Dietary and Lifestyle Behaviors and Weight Status and Their Self-Perceptions among Health Sciences University Students in North Lebanon. Biomed. Res. Int..

[76] Pelletier J.E., Laska M.N. (2013). Campus Food and Beverage Purchases Are Associated with Indicators of Diet Quality in College Students Living off Campus. Am. J. Health Promot..

[77] Alfaro-González S., Garrido-Miguel M., Fernández-Rodríguez R., Mesas A.E., Bravo-Esteban E., López-Muñoz P., Rodríguez-Gutiérrez E., Martínez-Vizcaíno V. (2024). Higher Adherence to the Mediterranean Diet Is Associated with Better Academic Achievement in Spanish University Students: A Multicenter Cross-Sectional Study. Nutr. Res..

[78] Whatnall M.C., Patterson A.J., Burrows T.L., Hutchesson M.J. (2019). Higher Diet Quality in University Students Is Associated with Higher Academic Achievement: A Cross-Sectional Study. J. Hum. Nutr. Diet..

[79] Luchtman D.W., Song C. (2013). Cognitive Enhancement by Omega-3 Fatty Acids from Child-Hood to Old Age: Findings from Animal and Clinical Studies. Neuropharmacology.

[80] Salari N., Hosseinian-Far A., Jalali R., Vaisi-Raygani A., Rasoulpoor S., Mohammadi M., Rasoulpoor S., Khaledi-Paveh B. (2020). Prevalence of stress, anxiety, depression among the general population during the COVID-19 pandemic: A systematic review and meta-analysis. Glob. Health.

[81] Muthuraman K., Sankaran A., Subramanian K. (2024). Association Between Sleep-related Cognitions, Sleep-related Behaviors, and Insomnia in Patients with Anxiety and Depression: A Cross-sectional Study. Indian J. Psychol. Med..

[82] Goncalves A., Le Vigouroux S., Charbonnier E. (2021). University Students’ Lifestyle Behaviors during the COVID-19 Pandemic: A Four-Wave Longitudinal Survey. Int. J. Environ. Res. Public Health.

[83] Jakobsdottir G., Stefansdottir R.S., Gestsdottir S., Stefansson V., Johannsson E., Rognvaldsdottir V., Gisladottir T.L. (2023). Changes in health-related lifestyle choices of university students before and during the COVID-19 pandemic: Associations between food choices, physical activity and health. PLoS ONE.

